# Supplementary material for: Treatment-Interval Changes in Serum Levels of Albumin and Histidine Correlated with Treatment Interruption in Patients with Locally Advanced Head and Neck Squamous Cell Carcinoma Completing Chemoradiotherapy under Recommended Calorie and Protein Provision
Source: Cancers (Basel). 2022 Jun 24;14(13):3112. doi: 10.3390/cancers14133112 (PMC9264877; doi:10.3390/cancers14133112)
Supplement: Supplementary file 1 [file cancers-14-03112-s001.zip › cancers-1757744-supplementary.pdf]

Supplementary Information

Table S1. Clinicopathologic variables, biochemical and nutrition-inflammation data, anthropometric and body composition characteristics, serum HLOP metabolite and treatment-related factors of 44 patients with LAHNSCC completing CCRT course stratified by the status of CRP change during the treatment course

Variables expressed as Numbers (%) or mean±SD	ALL	ΔCRP increase§	ΔCRP decrease§	*p* value*
Included patient number	44 (100.0)	29 (65.9)	15 (34.1)	
Treatment interruption (no : yes)	35 (79.5) : 9 (20.5)	20 (69.0) : 9 (31.0)	15 (100.0) : 0 (0.0)	0.016*
Clinicopathologic				
Age (years)	54.7 ± 9.2	56.1 ±8.8	53.0 ± 9.6	0.189
≥65 : <65	6 (13.6) : 38 (86.4)	5 (17.2) : 24 (82.8)	1 (6.7) : 14 (93.3)	0.333
Sex (male: female)	42 (95.5) : 2 (4.5)	28 (96.6) : 1 (3.4)	14 (93.3) : 1 (6.7)	0.627
Tumor location (oral cavity/non-oral cavity)				0.754
Buccal mucosa/Tonsil	3 (6.8) / 3 (6.8)	2 (6.9) / 2 (6.9)	1 (6.7) / 1 (6.7)	
Tongue/Tongue base	12 (27.2) / 1 (2.2)	9 (31.0) / 0 (0.0)	3 (20.1) / 1 (6.7)	
Gingiva/Soft palate	5 (11.4) / 0 (0.0)	3 (10.3) / 0 (0.0)	2 (13.3) / 0 (0.0)	
Retromolar /Hypopharynx	2 (4.5) / 10 (22.7)	2 (6.9) / 7 (24.1)	0 (0.0) / 3 (20.0)	
Hard palate / Larynx	1 (2.2) / 5 (6.7)	0 (0.0) / 3 (10.3)	1 (6.7) 2 (13.3)	
Lip/--	2 (4.5) / --	1 (3.4) / --	1 (6.7) / ---	
TNM stage				0.191
III : IVA : IVB	5 (11.4) : 25 (56.8) : 14 (31.8)	5 (17.2):16 (55.2) : 8 (27.6)	0 (0.0) : 8 (53.3) : 7 (46.7)	
T status				0.359
T0-2 : T3-4	11 (25.0) : 33 (75.0)	6 (20.7) : 23 (79.3)	5 (33.3) : 10 (66.7)	
N status				0.939
N0-1 : N2-3	15 (34.1) : 29 (65.9)	10 (34.5) : 19 (65.5)	5 (33.3) : 10 (66.7)	
Histological differentiation grade				
well : moderately : poorly	6 (13.6): 32 (72.8) : 6 (13.6)	5 (17.2): 19 (65.6) : 5 (17.2)	1 (6.7): 13 (86.7) : 1 (6.7)	0.328
ECOG performance status (0 : 1 : 2)	3 (6.9) : 39 (88.6) : 2 (4.5)	2 (6.9) : 25 (86.2) : 2 (6.9)	1 (6.7) : 14 (93.3) : 0 (0.0)	0.579
Tracheostomy (no *vs.* yes)	27 (61.4) : 17 (38.6)	17 (58.6) : 12 (41.4)	10 (66.6) : 5 (33.4)	0.603
Smoking exposure (no *vs.* yes)	7 (15.9) : 37 (84.1)	5 (17.2) : 24 (82.8)	2 (13.3) : 13 (86.7)	0.737
Alcohol consumption (no *vs.* yes)	12 (27.3) : 32 (72.7)	8 (27.6) : 21 (72.4)	4 (26.7) : 11 (73.3)	0.948
Betel nut use (no *vs.* yes)	16 (36.4) : 28 (63.6)	11 (37.9) : 18 (62.1)	5 (33.4) : 10 (66.6)	0.764
HN-CCI (0 : ≥1)	0 (0.0) : 44 (100.0)	0 (0.0) : 29 (100.0)	0 (0.0) : 15 (100.0)	----
PG-SGA assessment before CCRT				
Malnutrition none: moderate : severe	9 (20.5): 26 (59.0) : 9 (20.5)	6 (20.7): 17 (58.6) : 6 (20.7)	3 (20.0): 9 (60.0) : 3 (20.0)	0.996
Biochemical data				
Before CCRT				
eGFR (mL/min/1.73 m²)	105.4 ± 28.8	104.8 ± 31.2	106.3 ± 24.5	0.878
ALT (U/L, normal ≤ 36)	22.5 ± 10.8	25.6 ±16.9	22.4 ± 9.4	0.516
Total bilirubin (mg/dL, normal ≤ 1.3)	0.8 ± 0.4	0.5 ±0.4	0.4 ± 0.1	0.434
Uric acid (mg/dL, normal < 7.0)	5.2 ± 1.7	5.2 ±1.7	5.1 ±2.1	0.885
Sugar (AC, mg/dL)	115.2 ± 16.5	111.1 ± 546.6	123.1 ± 51.8	0.445
Treatment-interval change (%)				
ΔeGFR%⁑	-11.3 ± 26.7	-6.6 ±29.5	-20.4 ±24.8	0.128
ΔALT%⁑	-22.5 ± 28.5	-18.0 ±18.8	-31.4 ±44.6	0.747
ΔTotal bilirubin%⁑	-2.2 ± 8.5	-1.0 ±11.6	-8.6 ±10.9	0.597

ΔUric acid%⁑	-3.5 ± 46.7	-11.2 ±7.4	12.5 ±6.9	0.038*
ΔSugar (AC)⁑	-9.1 ± 36.7	-7.4 ±46.8	-12.1 ±26.5	0.697
Anthropometric data and blood NIB data				
Before CCRT				
BW (kg)	63.9 ± 12.4	63.7 ± 11.4	64.3 ± 14.4	0.899
BMI (kg/m²)	23.1 ± 3.9	23.2 ± 3.7	22.8 ± 4.2	0.782
< 18.5 : ≥ 18.5	10 (22.7) : 34 (77.3)	7 (24.1) : 22 (75.9)	3 (20.0) : 12 (80.0)	0.594
Hb (g/dL)	12.0 ± 1.8	11.9 ± 1.4	12.0 ± 2.4	0.963
WBC (x10³ cells/mm³)	6.6 ±2.8	6.6 ±3.2	6.5 ±1.7	0.920
Platelet count (x10³/mm³)	260.1 ± 91.4	244.3 ± 82.2	290.6 ± 103.1	0.111
TLC (x10³ cells/mm³)	1.8 ±0.6	1.8 ±0.6	1.7 ± 0.7	0.761
< 1.5 : ≥ 1.5	14 (31.8) : 30 (68.2)	8 (27.6) : 21 (72.4)	6 (40.0) : 9 (60.0)	0.402
TNC (x 10³/mm³)	4.0 ±0.3	4.0 ±0.4	4.0 ± 1.2	0.941
TMC (x 10³/mm³)	0.5 ±0.3	0.5 ±0.3	0.4 ± 0.1	0.389
Albumin (g/dL)	3.9 ±0.4	3.9 ±0.4	3.9 ±0.3	0.827
< 3.5 : ≥ 3.5	5 (11.4) : 39 (88.6)	3 (10.3) : 26 (89.7)	2 (13.3) : 13 (86.7)	0.767
Prealbumin (g/dL, normal: 20–40)	25.3 ± 5.4	25.3 ± 5.7	25.4 ± 5.1	0.941
Transferrin (g/dL normal: 200–360)	208.0 ± 34.6	208.9 ± 36.3	206.2 ± 32.1	0.807
Total cholesterol (mg/dL, normal < 200)	169.3 ± 41.8	167.8 ± 96.5	172.4 ± 42.0	0.760
Triglyceride (mg/dL, normal < 150)	160.5 ± 15.4	152.5 ± 17.2	175.8 ± 116.3	0.483
CRP (mg/dL)	6.3 ± 7.7	3.5 ± 4.0	11.7 ± 10.2	0.009*
Treatment-interval change (%)				
ΔBW%⁑	-4.9 ±5.7	-6.2 ±5.9	-2.6 ± 4.6	0.041*
ΔBMI%⁑	-4.9 ±6.4	-6.5 ±6.4	-1.8 ± 5.2	0.018*
ΔHb%⁑	-13.3 ±14.1	-13.5 ±2.5	-12.6 ± 3.9	0.836
ΔWBC%⁑	-25.9 ±51.6	-20.8 ±10.8	-36.0 ± 18.6	0.359
ΔPlatelet%⁑	-15.5 ±37.8	-13.6 ± 40.4	-19.1 ± 32.9	0.654
ΔTLC%⁑	-24.8 ±8.5	-34.7 ± 32.6	-17.5 ± 21.4	0.211
ΔTNC%⁑	16.4 ±3.0	18.1 ±3.6	11.7 ± 5.6	0.249
ΔTMC%⁑	-4.0 ±10.9	-5.9 ± 11.7	-1.3 ± 23.3	0.813
ΔAlbumin%⁑	-1.0 ±14.1	-2.6 ± 15.2	6.8 ± 6.4	0.031*
ΔPrealbumin%⁑	-9.7 ±28.2	-9.7 ± 23.8	10.8 ± 31.7	0.020*
ΔTransferrin%⁑	-13.1 ±13.3	-6.2 ± 21.5	6.9 ± 20.4	0.041*
ΔCholesterol%⁑	0.9 ± 21.1	-1.2 ± 22.0	4.9 ± 19.3	0.366
ΔTriglyceride%⁑	-3.1 ±41.6	2.8± 43.3	-14.4 ± 36.7	0.198
ΔCRP%⁑	609.5 ±30.6	955.1 ± 454.9	-58.6± 7.9	0.034*
DXA-related measurements				
Before CCRT				
LBM (kg)	43.8 ± 5.9	43.8 ± 5.6	43.9 ± 6.6	0.925
TFM (kg)	17.5 ± 7.3	17.5 ± 7.0	17.7 ± 8.2	0.914
ASM (kg)	18.5 ± 3.3	18.6 ± 3.1	18.5 ± 3.9	0.969
Treatment-interval change (%)				
ΔLBM%⁑	-7.1 ±6.0	-9.2± 5.1	-3.6 ± 1.1	0.001*
ΔTFM%⁑	-3.4 ±13.2	-3.4± 20.2	-3.1 ± 18.1	0.908
ΔASM%⁑	-7.8 ±8.5	-10.8± 8.0	-2.0 ± 6.4	0.027*
HLOP Metabolites				
Before CCRT				
Histidine (μM)	78.8 ± 16.2	78.9 ± 15.4	78.4 ± 18.3	0..917
Leucine (μM)	128.7 ± 40.2	120.4 ± 36.5	144.9 ± 43.1	0.054

Ornithine (μM)	122.5 ± 35.4	122.3 ± 39.2	122.6 ± 27.8	0.983
Phenylalanine (μM)	63.1± 10.4	63.6 ± 16.4	65.5 ± 17.1	0.705
Treatment-interval change (%)				
ΔHistidine%⁑	-1.4 ±28.2	-6.2± 15.3	7.6 ± 11.5	0.038*
ΔLeucine%⁑	-7.5 ±27.2	-3.5± 24.0	-15.3 ± 23.4	0.076
ΔOrnithine%⁑	-7.0 ±23.4	-11.7± 27.6	-12.1 ± 22.9	0.963
ΔPhenylalanine%⁑	13.9 ±33.3	8.6± 28.4	5.9 ± 33.0	0.785
Mean daily calorie intake during CCRT †	26.9 ± 6.6	27.2 ± 7.1	26.5 ± 5.7	0.738
≥ 25 and < 30 : ≥ 30	31 (70.5) : 13 (29.5)	19 (65.5) : 10 (34.5)	12 (80.0) : 3 (20.0)	0.318
Mean daily protein intake during CCRT††	1.0 ± 0.3	1.0 ± 0.2	1.0 ± 0.3	0.751
Mean daily CHO intake during CCRT††	3.7 ± 0.9	3.7 ± 0.7	3.7 ± 0.9	0.738
Mean daily fat intake during CCRT††	0.8 ± 0.2	0.9 ± 0.2	0.8 ± 0..2	0.739
Feeding tube placement (no *vs.* yes)	22 (50.0) : 22 (50.0)	16 (55.2) : 13 (44.8)	6 (40.0) : 9 (60.0)	0.340
Mean days of feeding tube placement during CCRT	20.3 ± 3.7	19.6 ±4.7	21.7 ± 5.8	0.792
CCRT data				
Radiotherapy				
Dose (Gy)	66.6 ± 3.9	65.9 ± 3.9	67.7 ± 4.0	0.154
Fractions	32.6 ± 1.3	32.3 ± 1.3	32.9 ± 1.2	0.146
Duration (days)	48.4 ± 5.1	48.6 ± 5.8	47.8 ± 3.5	0.648
Cisplatin dose (mg/m^2)	233.4 ± 22.2	238.1 ± 14.6	215.0 ± 35.7	0.004*
Grade 3/4 toxicity during CCRT				
Non-hematologic				
Dermatitis (no *vs.* yes)	43 (97.7) : 1 (2.3)	28 (96.6) : 1 (3.4)	15 (100) : 0 (0.0)	0.608
Pharyngitis (no *vs.* yes)	42 (95.5) : 2 (4.5)	27 (93.1) : 2 (6.9)	15 (100) : 0 (0.0)	0.298
Infection (no *vs.* yes)	30 (68.2) : 14 (31.8)	20 (69.0) : 9 (31.0)	10 (66.7) : 5 (33.3)	0.877
Mucositis (no *vs.* yes)	33 (75.0) : 11 (25.0)	19 (65.5) : 10 (34.5)	14 (93.3) : 1 (6.7)	0.043*
Emesis (no *vs.* yes)	41 (93.2) : 3 (6.8)	27 (93.1) : 2 (6.9)	14 (93.3) : 1 (6.7)	0.977
Hematologic				
Anemia (no *vs.* yes)	41 (93.2) : 3 (6.8)	27 (93.1) : 2 (6.9)	14 (93.3) : 1 (6.7)	0.977
Neutropenia (no *vs.* yes)	29 (65.9) : 15 (34.1)	20 (69.0) : 9 (31.0)	9 (60.0) : 6 (40.0)	0.552
Thrombocytopenia (no *vs.* yes)	38 (86.4) : 6 (13.6)	25 (86.2) : 4 (13.8)	13 (86.7) : 2 (13.3)	0.801
Number of grade 3/4 toxicity	1.2 ± 1.1	1.4 ± 1.2	1.0 ± 1.0	0..338

†unit: Kcal /kg/day ††unit: g/kg/day

* Comparing the value difference between treatment interruption and treatment with no interruption for each variable; $p < 0.05$, statistically significant

§ ΔCRP increase was defined as a positive value obtained by subtracting the pre-treatment CRP value from the post-treatment CRP value. ΔCRP decrease was defined as a negative value obtained by subtracting the pre-treatment CRP value from the post-treatment CRP value.

The Mann–Whitney test was used for ΔHb%, CRP, Δ uric acid%, total bilirubin, mean days for feeding tube placement, dose, fraction and days of radiotherapy, cisplatin dose, all DXA-related measurements, all metabolites, and the number of grade 3/4 toxicities. Independent *t* test was used for other continuous variables. The chi-square test was used for all categorical data.

⁑Δ indicates the value obtained by subtracting the pretreatment value from the post-treatment value; % indicates

(Δ value/ the pretreatment value) x100%

Abbreviations: LAHNSCC, locally advanced head and neck squamous cell carcinoma; CCRT, concurrent chemoradiotherapy; SD, standard deviation; HN-CCI, Charlson comorbidity index; ECOG, Eastern Cooperative Oncology Group; CHO, carbohydrate; PG-SGA, patient-generated subjective global assessment; BW, body weight; BMI, body mass index; eGFR, estimated glomerular filtration rate; ALT, alanine aminotransferase; Hb, hemoglobin; WBC, white blood cell; TLC, total lymphocyte count; TNC, total neutrophil count; TMC, total monocyte count; CRP, C-reactive protein; DXA, dual-energy X-ray absorptiometry; LBM, lean body mass; TFM, total fat mass; ASM, appendicular skeletal mass; HLOP, histidine, leucine, ornithine, and phenylalanine.

Table S2. Associations of clinicopathologic variables, NIBs, body composition parameters, treatment-related factors, and serum metabolites with treatment interval changes of leucine and ornithine over the CCRT course in 44 patients with LAHNSCC completing CCRT

**Variables**	**ΔLeucine%**			**ΔOrnithine%**		
	Univariate	Multivariate		Univariate	Multivariate	
	p-value*	Coefficient (95% CI)	*p*-value*	*p*-value*	Coefficient (95% CI)	*p*-value*
Clinicopathologic factors						
Age	0.779			0.965		
Sex (male *vs* female)	0.803			0.803		
Tumor location (OC *vs* NOC)	0.859			0.281		
TNM stage (III *vs* IVA *vs* IVB)	0.602			0.068		
T status (T1-2 *vs* T3-4)	0.007*			0.376		
N status (N0-1 *vs* N2-3)	0.732			0.892		
Histologic grade (well *vs* moderately *vs* poorly)	1.00			1.00		
Smoking (no *vs* yes)	0.550			0.032*		
Alcohol (no *vs* yes)	0.067			0.271		
Betel nut (no *vs* yes)	0.884			0.079		
ECOG performance status (0 : 1 : 2)	0.163			0.547		
Tracheostomy (no *vs* yes)	0.206			0.138		
PG-SGA before CCRT (none : moderate : severe)	0.385			0.247		
Biochemical data						
**Before CCRT**						
eGFR (mL/min/1.73 m^2)	0.930			0.840		
ALT (U/L)	0.069			0.953		
Total bilirubin (mg/dL)	0.437			0.931		
Uric acid (mg/dL)	0.756			0.182		
Sugar (AC)((mg/dL)	0.810			0.112		
**Treatment-interval change (%)**						
ΔeGFR%⁑	0.640			0.237		
ΔALT%⁑	0.114			0.261		
ΔTotal bilirubin%⁑	0.066			0.334		
ΔUric acid%⁑	0.573			0.495		

ΔSugar (AC)⁑	0.745			0.783		
Anthropometric data and blood NIBs data						
**Before CCRT**						
BW (kg)	0.441			0.850		
BMI (kg/m^2)	0.466			0.905		
Hb (g/dL)	0.016*			0.189		
WBC (x10^3 cells/mm^3)	0.025*			0.176		
Platelet count (x10^3/mm^3)	0.194			0.794		
TLC (x10^3 cells/mm^3)	0.036*	13.108 (6.210 ~ 19.990)	<0.001*	0.409		
TNC (x 10^3/mm^3)	0.086			0.151		
TMC (x 10^3/mm^3)	0.032*			0.368		
Albumin (g/dL)	0.113			0.720		
Prealbumin (g/dL)	0.072			0.425		
Transferrin (g/dL)	0.311			0.199		
Total cholesterol (mg/dL)	0.313			0.363		
Triglyceride (mg/dL)	0.475			0.892		
CRP (mg/dL)	0.486			0.498		
**Treatment-interval change (%)**						
ΔBW%⁑	0.747			0.597		
ΔBMI%⁑	0.387			0.510		
ΔHb%⁑	0.002*	0.470 (0.127 ~ 0.816)	0.009*	0.032*		
ΔWBC%⁑	0.630			0.185		
ΔPlatelet%⁑	0.092			0.756		
ΔTLC%⁑	0.179			0.414		
ΔTNC%⁑	0.307			0.876		
ΔTMC%⁑	0.508			0.923		
ΔAlbumin%⁑	0.305			0.146		
ΔPrealbumin%⁑	0.769			0.162		
ΔTransferrin%⁑	0.658			0.103		
ΔCholesterol%⁑	0.021			0.003	0.430 (0.054 ~ 0.807)	0.026*
ΔTriglyceride%⁑	0.343			0.821		

ΔCRP%⁑	0.951			0.053
DXA-related measurements				
**Before CCRT**				
LBM (kg)	0.694			0.664
TFM (kg)	0.385			0.990
ASM (kg)	0.920			0.917
**Treatment-interval change (%)**				
ΔLBM%⁑	0.388			0.633
ΔTFM%⁑	0.326			0.105
ΔASM%⁑	0.849			0.915
HLOP Metabolites				
**Before CCRT**				
Histidine (μM)	<0.001*			0.052
Leucine (μM)	<0.001*	-0.462 (-0.536 ~ -0.309)	<0.001*	0.545
Ornithine (μM)	0.001*			0.005*
Phenylalanine (μM)	<0.001*			0.303
**Treatment-interval change (%)**				
ΔHistidine%⁑	0.015*			0.022*
ΔLeucine%⁑	----			0.004*
ΔOrnithine%⁑	0.004*	0.265 (0.082 ~ 0.448)	0.006*	----
ΔPhenylalanine%⁑	0.001*			0.102
Mean daily calorie intake during CCRT †	0.764			0.697
Mean daily protein intake during CCRT††	0.723			0.409
Mean daily CHO intake during CCRT††	0.864			0.029
Mean daily fat intake during CCRT††	0.959			0.686
Feeding tube placement (no vs. yes)	0.583			
Mean days of feeding tube placement during CCRT	0.750			0.083
CCRT factor				
RT dose (Gy)	0.710			0.647
RT fractions	0.296			0.851
RT duration (days)	0.439			0.709

Cisplatin dose (mg/m^2)	0.153	0.119
CCRT-induced grade 3/4 toxicity		
**Non-hematologic**		
Dermatitis (no *vs* yes)	0.318	0.091
Pharyngitis (no *vs* yes)	0.013*	0.211
Mucositis (no *vs* yes)	0.979	0.391
Infection (no *vs* yes)	0.880	0.705
Emesis (no *vs* yes)	0.563	0.596
**Hematologic**		
Anemia (no *vs* yes)	0.221	0.394
Neutropenia (no *vs* yes)	0.594	0.450
Thrombocytopenia (no *vs* yes)	0.777	0.777

*indicates a significant p-value < 0.05.

Univariate analysis: simple linear regression model for all continuous variables; Mann–Whitney test for sex, T status, N status, smoking, alcohol, betel nut, tracheostomy, and feeding tube placement; Kruskal–Wallis test for TNM stage, histological grade, ECOG performance status, and PG-SGA

⁑Δ indicates the value obtained by subtracting the pretreatment value from the post-treatment value; % indicates (Δ value/ the pretreatment value) x100%

Abbreviations: NIBs, nutritional and inflammatory biomarkers; LAHNSCC, locally advanced head and neck squamous cell carcinoma; CCRT, concurrent chemoradiotherapy; CI, confidence interval; OC, oral cavity; NOC, non-oral cavity; TNM, tumor node metastasis; ECOG, Eastern Collaboration Oncology Group; RT, radiotherapy; PG-SGA, patient-generated subjective global assessment; eGFR, estimated glomerular filtration rate; ALT, alanine transaminase; NIBs, nutritional/inflammatory biomarkers; BMI, body mass index; BW, body weight; Hb, hemoglobin; WBC, white cell count; TLC, total lymphocyte count; TNC, total neutrophil count; TMC, total monocyte count; CRP, C-reactive protein; DXA, dual-energy X-ray absorptiometry; LBM, lean body mass; TFM, total fat mass; ASM, appendicular skeletal mass; HLOP, histidine, leucine, ornithine, and phenylalanine.

Table S3. Clinicopathologic variables, biochemical and nutrition-inflammation data, anthropometric and body composition characteristics, and serum HLOP metabolite of 50 patients with LAHNSCC stratified by the status of complete and incomplete CCRT

**Variables expressed as Numbers (%) or mean±SD**	**Complete CCRT**	**Incomplete CCRT**[§]	***p* value***
Included patient number	44 (88.0)	6 (12.0)	
Clinicopathologic			
Age (years)	54.7 ±9.1	56.5 ± 7.4	0.645
Sex (male: female)	42 (95.5) : 2 (4.5)	6 (100.0) : 0 (0.0)	0.594
Tumor location (oral cavity/non-oral cavity)			0.133
Buccal mucosa/Tonsil	3 (6.8) / 3 (6.8)	1 (16.7) / 1 (16.7)	
Tongue/Tongue base	12 (27.2) / 1 (2.2)	1 (16.7) / 0 (0.0)	
Gingiva/Soft palate	5 (11.4) / 0 (0.0)	1 (16.7) / 0 (0.0)	
Retromolar /Hypopharynx	2 (4.5) / 10 (22.7)	0 (0.0) / 1 (16.7)	
Hard palate / Larynx	1 (2.2) / 5 (6.7)	0 (0.0) / 1 (16.7)	
Lip/--	2 (4.5) / --	0 (0.0)/ ---	
TNM stage			0.149
III : IVA : IVB	4 (9.1):22 (50.0) : 18 (40.9)	0 (0.0) : 1 (16.7) : 5 (83.3)	
T status			0.849
T0-2 : T3-4	13 (29.5) : 31 (70.5)	2 (33.3) : 4 (66.7)	
N status			0.941
N0-1 : N2-3	14 (31.8) : 30 (68.2)	2 (33.3) : 4 (66.7)	
Histological differentiation grade			
well : moderately : poorly	5 (11.4): 32 (72.7) : 7 (15.9)	1 (16.7) : 5 (83.3) : 0 (0.0)	0.583
ECOG performance status (0 : 1 : 2)	3 (6.8) : 39 (88.6) : 2 (4.5)	2 (33.3) : 4 (66.7) : 0 (0.0)	0.358
Tracheostomy (no *vs.* yes)	27 (61.4) : 17 (38.6)	3 (50.0) : 3 (50.0)	0.594
Smoking exposure (no *vs.* yes)	7 (15.8) : 37 (84.1)	1 (16.7) : 5 (83.3)	0.962
Alcohol consumption (no *vs.* yes)	12 (27.3) : 32 (72.7)	2 (33.3) : 4 (66.7)	0.756
Betel nut use (no *vs.* yes)	11 (37.9) : 18 (62.1)	3 (50.0) : 3 (50.0)	0.519
HN-CCI (0 : ≥1)	18 (40.9) : 26 (59.1)	2 (33.3) : 4 (66.7)	0.722
PG-SGA assessment before CCRT			
Malnutrition none : moderate : severe	9 (20.5): 26 (59.0) : 9 (20.5)	0 (20.0): 4 (66.7) : 2 (33.3)	0.433
Biochemical data			
eGFR (mL/min/1.73 m^2)	104.2 ± 32.2	105.0 ± 29.5	0.910
ALT (U/L, normal ≤ 36)	24.6 ±14.7	33.7 ± 21.2	0.188
Total bilirubin (mg/dL, normal ≤ 1.3)	0.7 ±0.4	0.9 ± 1.0	0.502
Uric acid (mg/dL, normal < 7.0)	5.2 ±1.8	5.1 ±1.3	0.948
Sugar (AC, mg/dL)	115.2 ± 48.2	110.0 ± 34.2	0.799
Anthropometric data and blood NIB data			
BW (kg)	63.9 ± 12.3	64.6 ± 7.2	0.900
BMI (kg/m^2)	23.0 ± 3.9	21.9 ± 2.6	0.517
Hb (g/dL)	12.0 ± 1.7	11.6 ± 2.4	0.637
WBC (x10^3 cells/mm^3)	6.6 ±2.8	6.6 ±1.9	0.975
Platelet count (x10^3/mm^3)	260.1 ± 91.4	270.3 ± 71.0	0.794
TLC (x10^3 cells/mm^3)	1.8 ±0.6	1.6 ± 0.3	0.480
TNC (x 10^3/mm^3)	4.0 ±2.0	4.2 ± 1.9	0.855
TMC (x 10^3/mm^3)	0.5 ±0.1	0.4 ± 0.2	0.329
Albumin (g/dL)	3.9 ±0.4	3.8 ±0.8	0.576
Prealbumin (g/dL, normal: 20–40)	25.3 ± 5.4	23.5 ± 6.6	0.249
Transferrin (g/dL normal: 200–360)	208.0 ± 34.6	196.2 ± 412.1	0.747

Total cholesterol (mg/dL, normal < 200)	169.3 ± 41.8	175.7 ± 54.2	0.736
Triglyceride (mg/dL, normal < 150)	152.5 ± 17.2	175.8 ± 116.3	0.483
CRP (mg/dL)	6.3 ± 7.7	10.4 ± 13.3	0.267
DXA-related measurements			
LBM (kg)	43.8 ± 5.9	46.7 ± 2.4	0.294
TFM (kg)	17.5 ± 7.3	15.6 ± 5.8	0.576
ASM (kg)	18.2 ± 3.7	18.4 ± 3.2	0.911
HLOP Metabolites			
Histidine (μM)	78.8 ± 16.2	76.7 ± 17.4	0..770
Leucine (μM)	128.7 ± 40.2	120.7 ± 29.5	0.641
Ornithine (μM)	122.4 ± 35.4	118.6 ± 26.4	0.802
Phenylalanine (μM)	64.3 ± 16.5	61.4 ± 17.3	0.698

* Comparison of the value difference between complete CCRT and incomplete CCRT for each variable; $p < 0.05$, statistical significance.

§ Incomplete CCRT group included drop-outs and incomplete data collection.

The Mann–Whitney test was used for CRP, total bilirubin, all DXA-related measurements, and all metabolites. Independent *t* test was used for other continuous variables. The chi-square test was used for all categorical data.

Abbreviations: LAHNSCC, locally advanced head and neck squamous cell carcinoma; CCRT, concurrent chemoradiotherapy; SD, standard deviation; HN-CCI, Charlson comorbidity index; ECOG, Eastern Cooperative Oncology Group; PG-SGA, patient-generated subjective global assessment; BW, body weight; BMI, body mass index; eGFR, estimated glomerular filtration rate; ALT, alanine aminotransferase; Hb, hemoglobin; WBC, white blood cell; TLC, total lymphocyte count; TNC, total neutrophil count; TMC, total monocyte count; CRP, C-reactive protein; DXA, dual-energy X-ray absorptiometry; LBM, lean body mass; TFM, total fat mass; ASM, appendicular skeletal mass; HLOP, histidine, leucine, ornithine, phenylalanine.
